# Rapid/point-of-care syphilis testing in high-income countries: insights and implementation challenges from the authors’ experience in Canada

**DOI:** 10.1128/jcm.00622-25

**Published:** 2026-08-25

**Authors:** Ameeta E. Singh, Byron M. Berenger, Kevin Fonseca

**Affiliations:** 1 Department of Medicine, University of Alberta 215465, Edmonton, Alberta, Canada; 2 Alberta Precision Laboratories 113196 , Calgary, Alberta, Canada; 3 Department of Pathology and Laboratory Medicine, University of Calgary 574842, Calgary, Alberta, Canada; 4 Department of Microbiology and Infectious Diseases, University of Calgary 198998, Calgary, Alberta, Canada; Medical College of Wisconsin Pathology, Milwaukee, Wisconsin, USA

**Keywords:** syphilis, rapid test, point-of-care test, rapid/point-of-care test, high-income country, screening, implementation

## Abstract

Rapid/point-of-care tests (RPOCTs) for syphilis can significantly contribute to addressing the ongoing syphilis epidemic in high-income countries (HICs) for individuals facing barriers to traditional healthcare access and who may be less likely to return for follow-up appointments. By providing immediate diagnosis and treatment, RPOCT helps reduce disease progression and transmission to sex partners and from pregnant women to their fetus. This review offers an overview of the resurgence of syphilis in HICs and discusses the advantages and limitations of existing RPOCTs. We also share our experience with implementing RPOCT programs and emphasize the importance of successful implementation, which requires a strategic approach that considers RPOCT performance characteristics, local epidemiology, and the specific needs of each setting. Community members and site leaders should be involved in program planning and implementation to ensure they are rooted in the lived experiences and knowledge of those most impacted. Equally important is engaging policymakers, public health authorities, and laboratories to support program adoption and sustainability. Patient, healthcare provider, and laboratory acceptance of RPOCT is crucial when selecting test kits and planning program delivery. Ongoing monitoring and evaluation are essential to assess program effectiveness and allow for strategic adjustments based on feedback and outcomes. This continuous improvement process optimizes the impact of RPOCT initiatives and maximizes public health efforts. Further research is needed to explore existing and new technologies that can enhance the accuracy of diagnosing syphilis infections and cost-effectiveness studies to support their use.

## INTRODUCTION

Syphilis, caused by the spirochete *Treponema pallidum* subsp*. pallidum*, is primarily transmitted through sexual contact or vertically during pregnancy (1). The natural course of untreated syphilis progresses through four distinct stages: primary, secondary, latent, and tertiary. It can lead to devastating clinical outcomes, including neurological abnormalities and infant mortality in the case of congenital syphilis (CS).

Rapid/point-of-care tests (RPOCTs) for syphilis, also referred to as rapid diagnostic tests or near-patient testing, are diagnostic tools conducted at or near the site of patient care and typically performed by healthcare providers (HCPs) rather than laboratory personnel (2). Many syphilis RPOCTs have been developed for use in clinical settings and have been available worldwide for at least two decades (3). Most RPOCTs are treponemal-only, which can diagnose past and present infection but not the stage of syphilis, which usually requires non-treponemal tests in combination with clinical examination. Dual syphilis (both treponemal and non-treponemal) RPOCTs have become available, marking a significant shift in the diagnostic landscape as they potentially reduce the need for parallel laboratory testing. While RPOCTs have historically been used extensively in low- and middle-income countries where access to standard laboratory testing is limited, their adoption in high-income countries (HIC) has been limited. The recent resurgence of infectious syphilis in HIC provides a unique opportunity and urgent need to scale up the use of these tests, especially in underserved populations.

This review provides an overview of the current epidemiology of syphilis in HICs. It also discusses the availability, performance, impact, and implementation considerations of syphilis RPOCTs.

## EPIDEMIOLOGY OF SYPHILIS IN HIGH-INCOME COUNTRIES

Syphilis incidence has increased dramatically in recent years in several HICs, including the United States (U.S.), Canada, Europe, Australia, and New Zealand (NZ) (4–15). While the greatest increases have been observed among gay and bisexual men (gbMSM), some regions have observed rising rates among heterosexual persons and women of reproductive age, which has led to a resurgence in CS (Table 1).

**TABLE 1:** Rates, population characteristics, and geographic variation of infectious and congenital syphilis in select high-income countries^*a*^

Country	Time period	Overall change in cases/rates	Population change	Congenital cases	Geographical rates	Social/demographic factors
USA (6, 7)	2000– 2024	Increase in primary and secondary syphilis from 5,979 cases (2.1 per 100,000) in 2000 to 14,652 cases (17.7 per 100,000) in 2022, then decline to 11,859 (12.2 per 100,000) in 2024	Cases among females increased 112%	Increase to 3,941 cases (109.6 per 100,000) in 2024	South, Midwest, and West regions have the highest rates of primary and secondary syphilis; highest rates in South Dakota and New Mexico	Highest rates among American Indian/Alaska Native > African American > other non-White persons
Canada (8–11)	2018– 2023	Increase (double) in cases from 6,371 to 12,135 cases (30.5 per 100,000)	Tripling of female cases; since 2018, 177% increase among non-gbMSM population compared with 2% among gbMSM	1993–2022: 127-fold increase from 0.3 to 32.7 per 100,000	Highest rates in the central (prairie) provinces of Alberta, Saskatchewan, and Manitoba	Indigenous persons disproportionately represented
Europe (12)	2000– 2023	Decline from 2000 to 2007, increase from 2007 to 2012 then sharp rise after 2021; increase since 2010 greatest among gbMSM and more recently among heterosexual persons	Increase among women from 2022 to 2023; 2023, majority of females aged 20–33 years	Cases declined between 2010 and 2016 then increased ~10% between 2016–2013	2023: Bulgaria, Hungary, and Portugal reported 51% of cases	Pregnant women with syphilis: migrant backgrounds, multiple sex partners, substance use, partners at high risk for STBBI
Australia (13, 14)	2011– 2022	Progressive increase among women of childbearing age; 2025 (first 6 months): 3,153 cases		Progressive increase in cases; 2025 (first 6 months): 9 cases, four infant deaths	Highest in regional and remote areas of northern and central Australia; Northern Territory highest rate	Indigenous/Torres Strait Islander women 7 times higher than non-Indigenous women
New Zealand (4, 15)	2011– 2022	2019: gbMSM accounted for 2/3 of syphilis diagnoses (1,231 per 100,000); heterosexual persons: 0.5 per 100,000	2011–2016: rising infections among women	2011–2016: no cases; 2018–2022: 12 cases (9.4 per 100,000)	Highest number of cases in Auckland	75% of women with syphilis during pregnancy of NZ Maori and Pacific Island descent

^*a*^ gbMSM, gay and bisexual men who have sex with men; STBBI, sexually transmitted and blood borne infections; NZ, New Zealand.

The resurgence of syphilis, including CS, in these regions is a growing public health concern caused by social, structural, behavioral, and health inequity factors (9). In Europe, affected populations include pregnant women with migrant backgrounds, women engaging in risky behaviors like multiple sexual partners or drug use, and partners of individuals at high risk of sexually transmitted and blood-borne infections (STBBIs) (12). Healthcare organization and inadequate antenatal care, including insufficient syphilis testing, inadequate treatment after a positive test result, and syphilis infections acquired after an initial negative screening in pregnant individuals with no identified risk factors, are also contributing factors. Poverty, houselessness, and substance use are also associated with the spread of syphilis (15). In HIC such as NZ and Canada, despite universal healthcare systems, a disproportionate number of syphilis cases occur among Indigenous persons and in areas of socioeconomic disadvantage. For example, in NZ, Indigenous Māori experience rates of syphilis during pregnancy that are significantly higher than non-Indigenous populations (15). Similarly, in Canada, the highest rates of syphilis have been noted in the prairie provinces, where a larger proportion are Indigenous people who face greater socioeconomic challenges (10, 11). Some of the reasons cited for these findings are fear and mistrust of the healthcare system due to stigma and discrimination, intergenerational trauma, and the impacts of colonization (5).

The World Health Organization (WHO) aims to reduce CS to ≤50 cases per 100,000 live births by 2030 in 80% of countries (16). The European region has set interim targets of ≤10 congenital syphilis cases per 100,000 live births in 2025 and ≤1 case per 100,000 live births in 2030 (17). While the EU/European Economic Area (EEA) overall has met the 2025 target and most countries have low vertical transmission, gaps in prevention exist in several countries reach the 2030 target. Australia and NZ aim to eliminate CS (defined as sustained zero CS cases) by 2030 (14, 18). The Americas have committed to eliminating syphilis and CS as a public health problem by 2030, with a target of reducing the incidence of CS to <0.5 cases per 1,000 live births and a 90% reduction from 2018 (19, 20). However, incident cases of syphilis are currently increasing in the Americas.

## SYPHILIS DIAGNOSTICS

### Direct and serology-based testing algorithms

The diagnosis and staging of syphilis is complex and is typically made using a combination of history, physical findings, past history of syphilis, and the results of direct detection and serologic tests (treponemal tests, TT, and non-treponemal tests, NTT) (3, 21). The *in vitro* culture of *T. pallidum* has only recently been achieved, but it remains technically challenging and is not yet available for routine clinical use (22). Historically, direct tests have included darkfield microscopy and immunohistochemistry, using specimens collected from lesions of primary or secondary syphilis (21, 23). These tests have largely been replaced by nucleic acid amplification tests (NAATs), with the highest sensitivities (72%–95%) reported from primary lesions of syphilis and lower sensitivities (20%–86%) from secondary syphilis lesions (21, 23).

In most HICs, syphilis is primarily diagnosed using laboratory-based serologic tests on automated platforms, which detect either immunoglobulin G (IgG) and/or immunoglobulin M (IgM) antibodies against either *T. pallidum* antigens (TT) or antibodies against non-treponemal antigens (mainly cardiolipin) that are produced by the host in response to a syphilis infection (NTT) (21, 24). Two laboratory-based syphilis screening algorithms are used worldwide: (i) traditional algorithm, which uses an NTT for screening and, if reactive, is followed by one or more TT for confirmation, and (ii) reverse algorithm, which uses a TT as the initial screen and, if reactive, is followed by an NTT and, for discordant results, a second TT (3, 21). A confirmatory TT is also used in some versions of the reverse algorithm, regardless of the NTT result (25). The Rapid Plasma Reagin (RPR) is an NTT that can be quantified to produce a titer that is used to help stage infection, identify re-infection, and follow treatment response (21).

### Overview of syphilis RPOCT and comparison with laboratory-based tests

The majority of syphilis RPOCT are qualitative TT and utilize lateral flow or vertical flow technology with embedded *Treponema* antigens, whereas laboratory-based TT are usually chemiluminescent immunoassays for screening, with confirmation by particle agglutination or line immunoblot (21). RPOCT also differ from laboratory-based serologic tests in several ways. (i) Specimen collection and type: most syphilis RPOCTs use fingerstick whole blood, while laboratory-based tests typically require venipuncture. (ii) Time to test result: since samples do not usually need to be transported to the laboratory, RPOCT results are usually available in under 30 minutes, while laboratory-based results typically take hours to days. (iii) Differences in performance: syphilis RPOCTs usually have lower sensitivities and specificities when compared with laboratory TT and NTT, especially at lower or non-reactive RPR titers (26). (iv) Type of result: most syphilis RPOCTs yield a qualitative result, but in some clinical circumstances, a quantitative NTT (usually only available through laboratory-based tests) is desirable. (v) Type of test administrator: most syphilis RPOCTs are performed by health professionals, while laboratory tests require laboratory technicians. (vi) Instrumentation: RPOCTs typically use simple and affordable test kits, whereas laboratory testing requires expensive and complex equipment.

The WHO has developed the ASSURED criteria for RPOCT: affordable by those at risk of infection, sensitive (few false negatives), specific (few false positives), user-friendly (simple to perform and requires minimum training), rapid (to enable treatment at first visit), robust (does not need refrigerated storage), equipment-free (no additional equipment needed), and delivered to those who need it (27). In 2019, the criteria were expanded to the REASSURED criteria, adding real-time connectivity, where tests and/or readers are used to power the reaction and/or read the test results and to provide required data to decision makers, and ease of specimen collection, adding that tests should be designed for use with non-invasive specimens (28). Although most syphilis RPOCTs meet some of these criteria, real-time connectivity is lacking, and although test kits include easy-to-use fingerstick devices, specimen collection may still be a hurdle.

### Types of syphilis RPOCTs

In 2023, the WHO summarized syphilis RPOCT, of which 18 are available on the global market and are summarized in Table 2 (24). The sensitivity and specificity for each test, with 95% confidence intervals (CIs), are as reported by the manufacturer on their website and when compared with a laboratory-based treponemal test, e.g., *Treponema pallidum* particle agglutination or hemagglutination (TPPA or TPHA) (where information provided) (29–46). Several reviews have also summarized the data on syphilis only (TT and TT/NTT tests) and dual syphilis/HIV RPOCT (47–51).

**TABLE 2:** Summary of syphilis rapid/point-of-care single and dual tests^*a*^

Test name	Manufacturer	Test methodology and test kit type	Time to test result (min)	Antigen used	Type of antibody detected	Specimen	Reference test(s) for syphilis	Sensitivity of syphilis tests (%, 95% CI)	Specificity of syphilis tests (%, 95% CI)
Single syphilis (TT) test									
Accu-Tell Syphilis Rapid Test (29)	AccuBiotech Co. Ltd (Beijing, China)	Lateral flow/strip	5	NS	IgG/IgM	WB, S, P	TPPA	99.9, 97.7–100	99.7, 98.2–100
Determine Syphilis TP (30)	Abbott Laboratories (Green Oaks, IL, USA)	IC	15	rTp 47	IgG/IgM	S, P, B	TPPA (TPHA)	97.73 (94.32)	100 (100)
First Response Syphilis Anti-TP (31)	Premier Medical Corporation, Gujarat, India	IC	20–25	NS	IgG/IgM	S, P, B	CLIA/TPPA/RPR	100	100
Onsite Syphilis Ab Combo Rapid Test (32)	CTK Biotech (San Diego, CA, USA)	Lateral flow chromatography	15	rTp	IgG/IgM/IgA	S, P, B	TPPA	100, 97.5–100	100, 97.5–100
Reveal TP (33)	MedMira Inc, Halifax, Nova Scotia, Canada	Vertical flow IC/cassette	Immediate	rTp Ag	NS	S, P, B	NS	100	100
Bioline Syphilis 3.0 (34)	Abbott Laboratories (Green Oaks, IL, USA)	IC/cassette	5–20	rTp 15, 17	IgG/IgM/IgA	S, P, B	TPHA	99.3	99.5
Syphicheck-WB (35)	The Tulip Group/Qualpro (Goa, India)	IC/cassette	15	rTp 17, 47	IgG/IgM	S, P, B	TPHA/Syphilis Mixed Titer Performance Panel (PSS202), Boston Biomedica Inc., USA	95.3	93.7
Syphilis Health Check (36)	Trinity Biotech (Bray, Ireland)	IC/cassette	10–15	rTp	IgG/IgM	S, P, B	Multiple including RPR/TPPA/TPHA/FTA-ABS	98	97.2
Syphilis Ab Rapid C (37)	Cypress Diagnostics (Hulshout, Belgium)	IC/cassette	15–20	rTp, 15, 17, 42, 47	IgG/IgM/IgA	S, P, B	TPPA	98.7, 92.2–99.8	98.8, 93.3–99.8
VisiTect Syphilis (38)	Omega Diagnostics (Alva, UK)	IC/cassette	30	rTp Ag	IgG/IgM	S, P, B	VDRL/TPHA/FTA-ABS	98	100
Dual syphilis (TT/NTT) tests									
DPP Syphilis Screen and Confirm^*b*^ (39)	Chembio Diagnostic Systems, Inc (Medford, NY)	Solid phase IC	15 min	rTp 17, cardiolipin	IgG/IgM	S, P, B, V	EIA/RPR/TPPA	TT component: 96.5 NTT component: 95.5	TT component: 99.7 NTT component: 98.6
Multiplex syphilis/HIV tests									
DPP HIV-Syphilis (40)	Chembio Diagnostic Systems, Inc (Medford, NY)	Solid phase IC/cassette	15	rTp 17	IgG/IgM	P, B	EIA/RPR/TPPA	94.7 MSM (52) 78.6 80.1 (MR)	95.5 MSM (52) 99.5 99.0 (MR)
First Response HIV1+2/Syphilis Combo Card Test (41)	Premier Medical Corporation Private Limited	IC	15–25	rTP 15, 17, 45, 47	IgG/IgM	S, P, B	CLIA/TPPA	100	100
INSTI HIV/Syphilis Duo Antibody Test (42)	bioLytical Laboratories (Richmond, BC, Canada)	Immunofiltration/cassette	1	rTp 17, 47	IgG/IgM	S, P, B	EIA/RPR/TPPA	Fingerstick 99.3%–100%	Fingerstick 99–99.8
Multiplo Rapid TP/HIV Test (43)	MedMira Inc (Halifax, Nova Scotia, Canada)	Vertical flow immunofiltration/cassette	3	rTp 15, 17, 47	IgG/IgM	S, P, B	EIA/RPR/TPPA	81–94.6	94.2–100
Onsite HIV/Syphilis Ab Combo Panel Rapid Test (44)	CTK Biotech (San Diego, CA, USA)	Lateral flow chromatography	15	rTp	IgG/IgM/IgA	S, P, B	TPPA	100	99.7
Bioline HIV/Syphilis Duo (45)	Abbott Laboratories (Green Oaks, IL, USA)	Solid phase IC/cassette	15–20	rTp 17	IgG/IgM/IgA	S, P, B	TPHA	99.9 MSM (53): 73.9	99.7 MSM (53) 99.6
Standard Q HIV/Syphilis Combo (46)	SD Biosensor, Inc (Gyeonggi-do, Korea)	Lateral flow IC	15–20	NS	NS	S, P, B	CLIA/TPPA	98.8, 97.1–99.5	100, 99.8–100

^*a*^ IC, immunochromatography; NS, not specified; S, serum; P, plasma; B, whole blood; TT, treponemal test; NTT, non-treponemal test; rTP, recombinant *Treponema pallidum* antigen; MR, Micro Reader; V, venous whole blood; CLIA, chemiluminescence immunoassay; RPR, rapid plasma regain; TPPA *Treponema pallidum* particle agglutination; TPHA, *Treponema pallidum* haemagglutination; VDRL, Venereal Disease Research Laboratory.

^*b*^ Requires handheld digital reader (DPP MicroReader).

#### Single syphilis (TT) RPOCT

Ten RPOCTs for syphilis are TT, which detect solely treponemal antibodies, and only one test (MedMira Reveal TP) provides an extremely rapid qualitative result in under 5 min (33).

#### Dual syphilis (TT/NTT) RPOCT

Only one dual syphilis (TT/NTT), the Chembio DPP Syphilis Screen and Confirm Assay, is in wide use. Contrary to one of the WHO ASSURED criteria (equipment-free), this test requires a Micro Reader to interpret the lateral flow results, which attempts to reduce inter- and intra-observer variability and is not affected by available lighting and operator experience (39). Although the use of a reader can, in theory, reduce variability in reading, the initial validation showed no difference between visual and electronic reading, and they have the disadvantage of needing to be replaced after 3,000 reads and requiring the purchase, deployment, and inventory of equipment and either batteries or an electrical connection.

#### Multiplex syphilis/HIV tests

The remaining seven tests are multiplex syphilis (TT only) and HIV tests (Table 2).

All the HIV components of multiplex syphilis and HIV test RPOCTs are designed to detect antibodies to both HIV-1 and HIV-2 but do not identify HIV antigens or RNA. In acute HIV seroconversion, HIV p24 antigen and HIV RNA are dominant before the appearance of antibodies, which can result in a false-negative finding in RPOCTs. Earlier RPOCT studies used less sensitive HIV diagnostic algorithms as the reference standard for diagnosis, e.g., a systematic review in 2017 reported that the reference standard varied from two to three RPOCTs confirmed by two EIAs, EIA only, and EIA plus Western Blot (49). Studies published since then have compared HIV RPOCT performance with more contemporary HIV laboratory-based algorithms (as available in the study’s country) and varied from screening with third- or fourth-generation HIV Ag/Ab screening assays and confirmation with either Western Blot or nucleic acid tests or two RPOCTs as the reference standard (52–56). The sensitivity of the HIV component of the dual RDT varies from 85% to 100% depending on the population tested and reference standard, with specificity generally in the high range (98%) (49). The WHO recommends a minimum sensitivity of 99% and specificity of 98% for HIV RDTs (57). While some RPOCTs meet these benchmarks, others struggle, particularly in terms of sensitivity. This shortfall may affect the appropriateness of some dual RDTs in settings where confirmatory testing may not be readily available or when persons are less likely to return for follow-up visits (57).

### Syphilis RPOCT performance data

The performance of syphilis RPOCT varies depending on several factors, including specimen type, specific test brand, syphilis stage, type of syphilis test (TT only versus TT/NTT), and population studied.

The specimen types used by syphilis RPOCTs (TT only or TT/NTT) include whole blood (including fingerstick), serum, or plasma specimens, with performance varying by specific test platform and specimen type (7). A meta-analysis found that sensitivity is slightly lower with whole blood versus serum; sensitivity ranged from 74%–90% for serum versus 74%–87% for whole blood, while specificity was 94%–99% and 96%–100%, respectively (58). The improved performance of serum samples is likely because they contain a higher concentration of biomarkers and lack the interfering substances present in whole blood (58). The performance between specimen types also varies by specific test brand. For example, for the Determine test, sensitivity was 90% with serum versus 86% with whole blood, as compared with the SD Bioline, which showed similar performance for serum and whole blood (87% and 85%, respectively) (58).

Performance varies by syphilis stage, which is a vital gap with current RPOCT. Sensitivity is significantly higher in cases of “active syphilis” (e.g., RPR titers > 1:8 dilutions) (52, 53, 59). With RPR titers of >1:8 dilutions, sensitivities are 92%–100%, compared with 28%–93% when the RPR is either non-reactive or <1:8 dilutions. The main disadvantage of this is that early cases may be missed, as the RPR may be non-reactive or <8 dils. Dual syphilis (TT and NTT) RPOCTs are theoretically capable of distinguishing between active and past infection (60). However, their accuracy in this regard remains to be determined, especially considering their lower sensitivity with low (<1:8 dilutions) RPR titers. One combination TT/NTT is currently available on the global market (Chembio DPP Syphilis Screen and Confirm Assay) (39). A meta-analysis of this test in 2016 reported overall agreement between the DPP test and reference serology of 85.2% (95% CI 84.4%–86.1%), with higher sensitivity with RPR titers of >1:16 dilutions (51). However, subsequent field studies have indicated that this test may result in significant underdiagnosis and undertreatment (up to 48%) of active syphilis among MSM in Italy and pregnant women in Burkina Faso, raising questions about the impact and cost-effectiveness of combined TT/NTT syphilis RPOCT versus TT-only syphilis RPOCT (59, 61). In the Italian study, the performance of the Chembio test was compared head-to-head with a TT-only syphilis RPOCT (SD Bioline 3.0 test), and the TT-only RPOCT demonstrated better sensitivity (e.g., 63.1%–91.6% with the TT-only test on serum compared with 36.9%–76.6% with the TT/NTT test) (59). More recently, a newer investigational dual syphilis (TT/NTT) device (MedMira Multiplo Complete Syphilis (TP/nTP) antibody test, MedMira Inc, Halifax, Canada) showed promising results in an STI clinic population, with an overall sensitivity (for any RPR titer) of 82.5% and specificity of 99.1%, increasing to a sensitivity of 94.1% for RPR dilutions ≥ 1:8 dilutions (62).

A systematic review noted that differences in testing protocols, training, and specimen collection are potential factors explaining the reduced performance in RPOCT between laboratory and real-world settings, emphasizing the need for appropriate training and quality assurance for operators (63)

### Incorporation of syphilis and HIV RPOCT into testing pathways

The U.S. National Syphilis and Congenital Syphilis Syndemic Federal Task Force recommends integrating syphilis (treponemal) RPOCT as the initial screening test into a reverse algorithm. If the RPOCT is used for initial screening for syphilis and is negative, further testing is not required unless the patient has recent exposure or symptoms of early syphilis (in which case, re-test in 2–4 weeks) (26). However, for best clinical care and given that the sensitivity of syphilis RPOCT is generally lower than laboratory-based tests, negative RPOCT should ideally be followed by laboratory-based serologic tests to help limit and evaluate false-negative results (26). A reactive RPOCT must be reflexed to a laboratory-based, quantitative NTT to determine if the infection is active and a second TT for confirmation.

For HIV, the U.S. Centers for Disease Control and Prevention (CDC) recommends the use of RPOCT for HIV to make a preliminary diagnosis of HIV and that all reactive rapid tests be assessed with a laboratory-based Ag/Ab assay (64).

The WHO proposes different testing and management algorithms depending on the population being tested (65). In prenatal populations, HIV-syphilis dual RPOCT are recommended with a recommendation to treat pregnant women who test positive for syphilis and consideration being given to re-treatment with positive syphilis RPOCT in subsequent pregnancies. While empiric re-treatment in subsequent pregnancies might be suitable in resource-poor settings or where the risk of lost follow-up is high, it may not be appropriate in regions with standard laboratory testing and assured patient follow-up. In contrast, for populations at high risk, an NTT is recommended following a positive syphilis RPOCT and treatment if no prior treatment or if a positive NTT result is obtained. The algorithm recommends that pregnant people with a positive HIV RPOCT and populations at high risk have confirmatory HIV testing done.

## ADVANTAGES AND DISADVANTAGES OF SYPHILIS RPOCT IN HIC

The main advantage of using syphilis RPOCT in HIC is the ability to diagnose and treat syphilis in persons who have not previously been infected with syphilis in a single visit. Same-visit treatment can prevent disease progression and limit transmission to sex partners and from pregnant women to her unborn child. It also reduces the need for follow-up visits and minimizes losses to follow-up. Standard test results can vary from hours to weeks, depending on the distance from the testing lab and the specific algorithm used (26). This makes RPOCT in HIC particularly suitable for populations at high risk of loss to follow-up, such as those experiencing homelessness and/or incarceration, persons who use substances, and those living in rural or remote areas.

However, the main disadvantage of syphilis TT-only RPOCTs is their inability to distinguish between active and previously treated syphilis, due to the persistence of treponemal antibodies following adequate treatment. In addition, a reactive syphilis RDT using a TT only may be classified as a biologic false-positive reaction on laboratory algorithms and would not necessitate treatment.

In HIC, there’s likely a higher expectation for care, necessitating more accurate diagnoses and only providing necessary treatment. This is facilitated by standard laboratory tests, which may not be accessible in resource-limited settings (58). While dual syphilis (TT and NTT) RPOCTs have the potential to differentiate between previously treated and active syphilis infections, current data indicate their accuracy is insufficient to replace standard parallel laboratory testing. Therefore, for both TT-only and TT/NTT syphilis RPOCTs in HICs, parallel standard laboratory testing is still generally recommended for positive results to confirm the syphilis stage, determine the need for additional treatment, and partner notification. While there is a risk to treating those without syphilis, careful selection of high-risk individuals (see the Implementation section below) can mitigate this, especially since the risk of administering a single dose of penicillin G benzathine is minimal.

## SYPHILIS RPOCT FOR SELF-TESTING

The United States Food and Drug Administration (FDA) recently approved the first over-the-counter (OTC) treponemal test for syphilis, the First to Know Syphilis Test (NOWDiagnostics Inc.) (66). This test is a lateral flow device that uses fingerstick whole blood specimens and provides a preliminary result in 15 min (67). Reported sensitivity was 93.4%, and specificity was 99.5% in a multi-site, prospective study (68). The availability of this OTC test is particularly appealing for individuals who value privacy or convenience, as it can help address barriers, such as stigma and limited access to healthcare services. Individuals who have a history of syphilis, are unable to obtain or afford the test, understand and follow the instructions, and have access to appropriate care if the test is positive would be unsuitable for syphilis RPOCT. The manufacturer has partnered with other agencies to provide linkages to care through the provision of online resources and the option to link to a virtual care sexual health clinic (69).

## IMPACT OF SYPHILIS RPOCT IN UNDERSERVED POPULATIONS IN HIGH-INCOME COUNTRIES

Our center recently reported on an interrupted time series analysis (ITSA) evaluating the systematic scale-up of syphilis RPOCT in Alberta (70). TT syphilis RPOCTs were deployed across the province in areas experiencing a resurgence of infectious syphilis; positive RPOCT in persons not previously syphilis positive, those with symptoms of infectious syphilis, and sexual partners of infectious syphilis cases were offered same-visit treatment, and all RPOCT (positive and negative) were accompanied by parallel laboratory testing using a reverse algorithm. The ITSA found that provincial scale-up was associated with a 25% decrease in new syphilis positivity rates (0.22 per 100,000 population) (70). The impact was greater than during the initial regional implementation phase, when a 15% decline in rates was observed. The overall effect was more pronounced in the general population versus the prenatal population (15.9 vs 12.2%) and among males than females (16.0% vs 14.5%). A mathematical modeling study conducted in Canadian Arctic communities where syphilis incidence reached 44 per 1,000 among sexually active individuals suggests that implementing syphilis RPOCT could avert a cumulative 33% of new infections over 5 years and 37% over 10 years (71). The model also demonstrated that combining RPOCT testing with contact tracing could enhance these effects. Another mathematical modeling study evaluated the impact of scaling up syphilis testing, including RPOCT during a syphilis outbreak among remote Aboriginal communities in Australia (72). The investigators found that the increase in testing achieved at the beginning of the outbreak helped to stabilize the epidemic and averted a substantially worse outcome resulting from higher onward transmission.

## COST EFFECTIVENESS OF SYPHILIS RPOCT IN HIGH INCOME COUNTRIES

Few data are available on the cost-effectiveness of using syphilis RPOCT in high-income countries. One study conducted in Peru (classified as an upper middle-income economy [73]) with relatively low syphilis prevalence (~1%) concluded that the use of syphilis RPOCT was cost-effective in low-prevalence settings (74). This study compared the cost of syphilis RPOCT with laboratory-based RPR testing and reported costs per woman tested and treated with RPOCT at a maternity hospital of $2.70 and $369, respectively, compared with $3.60 and $740 for RPR. The cost per disability-adjusted life year (DALY) averted using RPOCT was $46 versus $109 for RPR. RPOCT showed lower costs compared with the WHO standard costs per DALY ($64). We anticipate that most HICs use reverse algorithms to screen for syphilis, which may increase the costs of testing. In addition, the cost of RPOCT is likely to be higher in HICs. Cost-effectiveness studies of syphilis RPOCT in HICs should ideally be region-specific and include the local epidemiologic data and laboratory, public health, and treatment costs in the calculations.

## IMPLEMENTATION CONSIDERATIONS AND CHALLENGES IN LOCAL HEALTHCARE SETTINGS: EXAMPLES FROM OUR EXPERIENCE

Several factors should be considered when deciding whether and how to implement RPOCT for syphilis. The rollout of syphilis RPOCT requires the use of implementation science to ensure success and should take into consideration the cultural, behavioral, socioeconomic, and health systems contexts (75–80). It also requires ensuring that it is implemented within an appropriate quality management system (QMS) for the deployment setting (81). There are many guides available for the implementation of RPOCT (75–80) and more specifically for syphilis RPOCT (27, 28, 75). Over the past 7 years, we have used these tests and learned several specific things that warrant further discussion (Table 3).

**TABLE 3:** Steps to consider when implementing a syphilis rapid/point-of-care testing program

Implementation strategy	Strategy specifics
1. Initial choice of tests and selection of deployment sites	1.1 Assessment of local surveillance data to identify target population(s) and geographic area(s): 1.2 Strategic deployment settings 1.3 Choice of test: patient and provider preference and acceptability a. Time to test result b. Type of specimen c. Syphilis only (TT or TT/NTT) vs multiplex (syphilis TT or syphilis TT/NTT with HIV) d. Operational characteristics of the test kit
2. Purchase and inventory: supply chain and cost considerations	
3. Organization	3.1 Linkages to care, including treatment, need for parallel standard laboratory testing, and reporting requirements 3.2 Engagement of policy and other decision makers 3.3 Community leadership and engagement
4. Personnel	Adequate provider training and support
5. Assessment and continual improvement: program oversight, monitoring, evaluation, and adaptation	
6. Equipment and safety: quality management systems	

### Initial choice of tests and selection of deployment sites

#### Assessment of local surveillance data to identify target population(s) and geographic area(s)

To deploy rapid diagnostic tests (RDTs) for syphilis, local surveillance data should be analyzed to identify affected populations and regions experiencing a resurgence. For example, after a syphilis outbreak was declared in Alberta in 2019 (82), local epidemiologic data revealed a significant increase in infectious syphilis cases in Edmonton and its surrounding areas. Many cases were concentrated in disadvantaged communities, such as those facing poverty, homelessness, and addictions. Indigenous persons, who represented 50% of cases despite representing only 7% of Alberta’s population, were disproportionately affected. About 20% of individuals did not return for test results after a standard blood draw. Review of cases from the previous year showed that over 70% of serum RPR tests had titers >1:8 dilutions, indicating active syphilis. This suggested a high success rate of identifying cases using a syphilis RPOCT device despite lower RPR titer detection limitations.

#### Strategic deployment settings

Since most current syphilis RPOCTs cannot distinguish between prior and current syphilis infection, the ideal settings HICs for syphilis RPOCT are those with low testing rates but likely high rates of syphilis in populations that are previously syphilis-naive. These settings may include areas where geographical barriers limit access to healthcare and testing, such as rural and remote areas, or in populations who face barriers to accessing healthcare, testing, or following up on test results (80).

The settings in which the tests were deployed were chosen in the Canadian Point-of-Care Tests for Syphilis and HIV (PoSH) study based on the areas with the highest syphilis rates (estimated prevalence ~10%) (52). Two extremely rapid (results in <5 min) syphilis/HIV readerless RPOCTs (INSTI HIV/Syphilis Duo Antibody Test, bioLytical Laboratories Inc, Vancouver, Canada, and Multiplo Rapid TP/HIV test, MedMira, Halifax, Canada) were evaluated compared with standard laboratory testing using a reverse algorithm for syphilis. At the start of the study, prior syphilis infection rates were very low. Sites or programs serving persons who were less likely to return for follow-up (such as persons experiencing incarceration, houselessness, and/or using substances) and in whom syphilis could potentially have the greatest impact (such as pregnant women) were selected. These settings included a provincial short-stay correctional facility, emergency departments (EDs) serving inner-city communities and indigenous communities, with 85% of all testing conducted in a short-stay (< 28 days) correctional facility and 85% of participants treated on the same day as the RPOCT. Since we intended to use the test kits for immediate treatment, we considered sites with HCPs who could perform and interpret the test upon approval by their licensing authority and administer treatment. In the province (Alberta) where the study was conducted, a provincial electronic medical record (EMR) enabled practitioners to determine whether individuals were previously positive on syphilis testing; syphilis RPOCT was only performed in persons who were previously syphilis negative. In our region, nurses and nurse practitioners conducted most of the tests, with a minority conducted by physicians. Following Health Canada (Canada’s federal agency equivalent to the U.S. Food and Drug Administration) approval of the tests, the use of syphilis RPOCT was scaled up across the province in the hospital EDs with on-site laboratories. To facilitate linkages to treatment, we also prioritized nurses working with provincial STI services.

#### Choice of test: patient and provider preference and acceptability

The choice of syphilis RPOCT test is influenced by patient and provider preferences, including the speed of test results, the required specimen type, whether the test detects syphilis alone or with HIV, and the test’s operational characteristics.

##### Time to test result

The time between specimen collection and test results delivery is crucial. Shorter wait times improve patient satisfaction and provider workflow efficiency. The extremely rapid TAT (<5 min) of the test kits in the PoSH study led to high patient and provider satisfaction and immediate treatment in ~85% of cases at the same visit (52). In a prior study in our region, lower provider and patient satisfaction was reported with another syphilis RPOCT (SD Bioline 3.0 Test, Standard Diagnostics, Inc., Korea), with results in approximately 20 min (83).

##### Type of specimen

Preferences vary depending on whether the test requires a fingerstick or venous blood draw specimen. When a venous blood draw can be used to conduct both the RPOCT and parallel laboratory tests, this is generally preferred by both patient and provider.

##### Syphilis only (TT or TT/NTT) vs multiplex (syphilis TT or syphilis TT/NTT with HIV)

The choice between syphilis only and multiplex tests affects provider workflow and patient acceptance, depending on the setting and population. Multiplex RPOCTs are preferred for convenience and operational simplicity and in prenatal and populations at high risk, are more efficient and cost-saving compared with separate screening programs (49, 84, 85). They also simplify training, reduce storage and transportation costs, and disposal (85). During the PoSH study, only a minority of participants preferred the syphilis-only test. However, other populations and settings may prefer syphilis-only testing. Wider screening with the RPOCT in acute care facilities faces ethical concerns about HIV testing and provider discomfort with pre- and post-test counseling, similar to reports in other EDs (86).

##### Operational characteristics of the test kit

The operational characteristics of the test affect providers’ preference and acceptability. A simpler RPOCT, with clear instructions for collecting fingerstick specimens, adding buffer accurately, and interpreting results, performs better (59). Reading POCT results manually for test kits without a reader is subjective and influenced by visual acuity, lighting, and operator experience (75). However, using a reader may not improve user acceptability or performance (53, 87). Thorough user acceptance is crucial. We experienced some difficulties with using the lancet and pipette provided with our original test kits (INSTI Multiplex HIV-1/HIV-2/Syphilis Antibody test and Multiplo Rapid TP/HIV test), but these issues were resolved with comprehensive training, reliable reference resources, and ongoing support.

Once the population and test are selected, there are numerous implementation considerations that must be considered, including the initiation and ongoing maintenance of a syphilis RPOCT program. We describe these using an adaptation of the Quality Systems Essentials Framework, which has been popularized for laboratory settings, aligns with and extends beyond Clinical Laboratory Improvement Amendments (CLIA) requirements, thus serving as a comprehensive quality management system that encompasses CLIA’s regulatory standards while incorporating best practices (88).

### Purchasing and inventory: supply chain and cost considerations

The planning phase for the PoSH study began before the COVID-19 pandemic disrupted global test kit supply, which may have affected cross-border trade. Fortuitously, Canadian manufacturers were chosen, reducing reliance on international supply chains and boosting resilience. Collaborative partnerships have improved point-of-care test accessibility in middle-income and low-income countries, lowering costs to less than $1.00 (89). However, similar partnerships in HIC are limited due to misconceptions about cost barriers to adoption. Cost remains a significant concern, especially for new programs requiring startup funding, kit costs, quality management, and ongoing monitoring and evaluation. In Alberta, centralizing kit ordering and distribution through the public health laboratory with the help of the centralized health provider procurement office proved beneficial based on funding models and local preferences.

### Organization

#### Linkages to care including treatment, need for parallel standard laboratory testing, and reporting requirements

The World Health Organization (WHO) recommends the “test and treat” approach in settings where syphilis is prevalent, particularly among pregnant women (90). This guidance highlights the necessity of establishing care pathways to ensure that individuals who test positive for syphilis receive timely treatment and appropriate follow-up. To deliver comprehensive care, it is essential that positive results from RPOCT are confirmed through standard laboratory testing, including quantitative NTT (26). This step determines whether the individual has an active infection and informs treatment decisions, follow-up care, and the notification of sexual partners. As outlined in the previous section, insufficient data are available on the accuracy of current dual syphilis (TT/NTT) RPOCT in distinguishing between previously treated and active infections and in accurately staging infections to discontinue the routine collection of parallel standard syphilis serology in HIC, especially for positive RPOCT. While current CDC and WHO management algorithms do not recommend routine collection of parallel standard laboratory tests for negative syphilis RPOCT, most testing locations in Canada encourage or require standard testing for all RPOCT, regardless of the result. This is because of the possibility of false negatives and because in our region, a negative result facilitates the connection to laboratory and public health electronic records to ensure patient follow-up. In our region, the increase in testing and positive results resulted in the need for additional partner notification services and an increase in parallel laboratory testing. These increased costs, which should be considered when evaluating cost-effectiveness. Additionally, records of positive RPOCT results and any treatments provided should be accessible to all healthcare providers involved in ongoing or follow-up care and must comply with public health reporting requirements. In our region, when RPOCT was conducted by providers in traditional health settings, the results were made available in the provincial EMR. However, results from testing conducted by community providers were not included. This posed surveillance and follow-up concerns, but these were mitigated by having parallel standard test results.

In recent years, the decline in the manufacturing and supply of penicillin G benzathine (BPG), the preferred treatment for syphilis, has presented challenges in providing syphilis testing and treatment within a single visit (91). In the United States, a shortage was initially reported in April 2023 and in Canada in December 2025 (92, 93). Both countries recently sourced an alternate formulation (Lentocilin S1200) from Portugal. In our region, existing supplies have been restricted for use in pregnant people and in persons at high risk of loss to follow-up. For individuals assessed to be able to adhere, oral doxycycline is prescribed twice daily for 14 days. Given the potential for RPOCT to lead to unnecessary treatment, this must be factored into the current BPG supply situation.

#### Engagement of policy and other decision makers

Despite the global availability of syphilis RPOCT for at least two decades, syphilis RPOCTs have not been deployed to maximal benefit in the same way as HIV and malaria RDTs (28). The main reasons for this are lack of advocacy and political will to translate the evidence to national policy, lack of funding, and lack of national control programs and strategies to coordinate efforts (28). Some countries and regions are starting to address this, but much work remains to be done to stem the tide of rising syphilis rates (14, 19).

Examples from other settings have demonstrated the importance of including policy makers from the beginning of a project and keeping them informed throughout the implementation to introduce RPOCTs within appropriate cultural and social contexts (94). By actively involving policy makers early in the process, stakeholders can ensure that the rollout of RPOCTs is aligned with local values, expectations, and regulatory frameworks. Ongoing communication with policy makers also facilitates smoother integration into existing health systems and increases the likelihood of sustained support and successful adoption.

Before the PoSH study in our area, we worked with key decision makers to help clinical staff use investigational devices in clinical settings. This allowed them to test and treat people with syphilis while also collecting data for companies to apply for Health Canada approval. Discussions with Health Canada about the urgent need to respond to the outbreak helped expedite the Investigational Testing Authorization (ITA) required to use the devices. The University of Alberta’s Health Research Ethics Board (HREB) played a crucial role in enabling clinical staff to participate in the research component.

#### Community leadership and engagement

As with other health programs and interventions, the deployment of syphilis RPOCTs should ideally be led by affected communities. Community leadership helps ensure that these interventions are meaningful, effective, and sustainable. When local stakeholders guide the process, initiatives are anchored in the knowledge and experience of those most impacted, which builds trust and strengthens relationships between providers and community members. Furthermore, community-led efforts are better positioned to address specific needs and priorities, resulting in greater relevance and responsiveness. This approach also helps foster a sense of ownership, contributing to long-term engagement and ongoing support for syphilis prevention and control measures.

The resurgence of syphilis in Alberta was first identified by public health authorities. Recognizing the importance of engaging those most affected, a local team reached out to the Alberta branch of Indigenous Services Canada (ISC-Alberta), the federal agency responsible for supporting indigenous communities across the province by providing essential health services. ISC-Alberta played a central role in facilitating meetings between the research team and leaders from impacted First Nations communities. These consultations were conducted prior to the launch of the PoSH study, ensuring that community perspectives and priorities were reflected in the planning and implementation process.

Advice received during these discussions led to meaningful modifications of the University of Alberta’s HREB protocols. Specifically, the protocols were amended to permit the use of verbal consent forms, as these were considered more culturally appropriate and acceptable to community members. The preference for verbal consent was based on the recognition that requiring signatures could evoke negative associations with colonization, making written consent forms less suitable in this context. Following the completion of the PoSH study, successes with rural deployment were seen due to further engagement of community leaders and integration into organizations facilitating community outreach.

### Personnel: adequate provider training and support

It is critical that POCT test operators receive adequate training before program implementation. Training materials should be developed in advance, and users should have opportunities to practice using the tests before applying them to patients. Comprehensive preparation not only builds operator confidence but also ensures the reliability of testing outcomes. In addition to technical proficiency, providers must be equipped to respond appropriately to positive syphilis or HIV test results. This often requires additional training and support in post-test counseling, especially for HIV, to guide patients through the next steps and reduce anxiety in the patient and provider (95). Recognizing that syphilis is a disease that has not been frequently covered in recent medical and nursing curricula, significant effort was invested prior to and during the PoSH study and following scale-up across the province to provide targeted training on syphilis epidemiology and clinical management. Since all syphilis RPOCTs are accompanied by standard laboratory tests in our province, the standard tests (including the serum RPR) are available to all providers with access to the provincial EMR. Access to expert consultation through provincial STI services and infectious diseases physicians was also prioritized, offering frontline staff timely guidance and support for complex cases. These training and support mechanisms are closely linked to improved care pathways and outcomes.

During scale-up of POCT in our province, an STI nurse was embedded in the inner-city ED. This role proved instrumental in navigating care pathways and facilitating both syphilis and HIV testing and led to a doubling of syphilis and HIV testing rates, increased use of syphilis/HIV RPOCT, and same-visit syphilis treatment initiation (96).

### Assessment and continual improvement: program oversight, monitoring, and evaluation, and adaptation

Effective program oversight is essential following the implementation of syphilis RPOCT initiatives. Ongoing monitoring and evaluation enable program administrators to systematically gather and assess data from patients, providers, and various clinical settings. This continuous feedback loop is crucial for identifying areas of strength as well as opportunities for improvement. Moreover, the ability to pivot and adapt the program in response to findings ensures that interventions remain relevant and responsive to the needs of those served. Engaging in regular evaluation and being receptive to feedback supports the sustained success and impact of syphilis RPOCT programs.

Following the completion of the PoSH study, findings from routine surveillance, as well as feedback and data collected during the study, informed a targeted province-wide expansion of syphilis testing. This scale-up was implemented in both acute care and community settings, with consideration given to the specific needs and context of each location. A preliminary evaluation of the performance of the INSTI Multiplex HIV/Syphilis RPOCT when conducted by laboratory techs at the onsite laboratories in seven acute care facilities found improved performance (*n* = 1,662, overall sensitivity of 88.2% and specificity of 99.8% for syphilis) when compared with the performance of the test by HCPs at the point of care in the PoSH study (*n* = 1,526, overall sensitivity 76.7% and specificity of 99.8% for syphilis) (52, 97). Despite this slight difference in performance, we facilitated the use of RPOCT at either on-site laboratories or directly by the healthcare provider at the point of patient care to ensure that testing could be integrated efficiently into existing workflows. The expansion followed further evaluation of test kits, such as comparing the performance of kits using fingerstick versus serum specimens (98). Upon receiving Health Canada approval, the program adopted added flexibility in specimen types; whole blood from either a fingerstick or venous blood draw or serum could be be used for RPOCT. This adjustment enabled broader applicability and responsiveness to local clinical needs, thereby enhancing the reach and effectiveness of syphilis testing across the province (97, 99).

### Equipment and safety: quality management systems (QMS)

QMS, including quality control (QC), assurance (QA), and documentation, are crucial for ensuring accurate and reproducible diagnostic laboratory testing results (100). Disciplines, notably Clinical Chemistry, that have extensively implemented POCTs devices both in hospitals and community settings, now have a well-developed infrastructure of QMS to support patient care. In contrast, for microbiology testing, almost all investigations are completed within laboratory facilities; therefore, a parallel POCT infrastructure is minimal and often modeled on the Clinical Biochemistry principles, which may not always be applicable or feasible when using these POCTs in the community. In our province, our provincial laboratory, public health, and chemistry departments collaborated to develop standardized procedures for QA/QC of syphilis/HIV RPOCT for laboratory and community settings.

Canada lacks national proficiency programs and guidelines for syphilis and HIV RPOCT, leading to inconsistencies in testing approaches across provinces. Therefore, operators must be trained on the sensitivity and specificity of POCTs, the importance of checking expiry dates, and monitoring storage conditions, to ensure that the kit integrity and reagents are maintained to ensure accurate results. This was ensured in our province through the development of QA/QC procedures for use in both acute care and community settings. Our provincial laboratory also conducted centralized lot release of the test kits, regular monitoring of the competency of the HCPs (similar to laboratory technologists), and followed up with operators and/or manufacturers when unusual RPOCT results or difficulties with the test kits arose. However, this was only possible because most funding came from research grants, which allowed for oversight under those grants. Since Alberta provincial authorities lack jurisdiction over community sites outside the provincial health systems, program oversight will be challenging once the grants are finished. This lack of ongoing infrastructure will likely ultimately hinder long-term prevention and control, patient management, and public health follow-up of syphilis.

## HOW LONG SHOULD SYPHILIS RPOCT PROGRAMS BE SUSTAINED?

Once implemented, data suggest that premature discontinuation of syphilis control programs could result in a rebound in rates (101). Mathematical modeling studies indicate that it is essential to maintain and even increase testing coverage in surrounding regions and communities, especially if there is incomplete testing/treatment of high-frequency transmitters and if population mobility is relatively high (71, 72, 100). If efforts diminish, the prevalence of infectious syphilis can quickly rebound, undermining previous progress in disease control.

Building on the successful scale-up of syphilis testing in Alberta, neighboring provinces have followed suit. The *Ayaangwaamiziwin* initiative—an Ojibwe term meaning “carefulness and preparedness”—will further expand syphilis and HIV RPOCT over the next three to five years throughout Alberta, Saskatchewan, and Manitoba, and adjacent territories (102).”

## AREAS FOR FUTURE RESEARCH

Improving the sensitivity of the TT component for single and dual syphilis and the NTT component of dual syphilis RPOCT for RPR titers <1:8 is needed. Further implementation science studies evaluating the feasibility and acceptability in different populations, by a broader range of testing providers, and in lower prevalence settings are also warranted. Studies should enroll adequate numbers of patients with different RPR titers and report on syphilis stages of infection, including re-infections. As noted in a recent systematic review of syphilis RPOCT, only one of nine included studies (the PoSH study [52]) included this information (47).

Given the limited data on cost-effectiveness in high-income countries, further health economic studies are necessary, especially if parallel laboratory testing for all syphilis RPOCT (positive and negative) is incorporated. It is unlikely that the recommendation for parallel testing will be discontinued in HIC until RPOCT performance matches standard testing algorithms. Cost effectiveness studies are critical to advocate for continued funding of current RPOCT programs in Canada (and likely other countries) as many are currently funded by time-limited grants.

Methods to enhance linkages to care, especially when syphilis RPOCT are used for self-testing or by non-HCP outside clinical settings, are needed to facilitate treatment and follow-up care. Current FDA-approved at-home testing instructs users to contact their healthcare provider or appropriate authority, but testing platforms that link to care, such as those that offer virtual visits upon a positive result, may be beneficial, similar to some at-home STI testing devices (103).

Finally, RPOCT incorporating NAAT tests and/or novel diagnostic markers for in-lab and RPOCT use also warrant further investigation to aid in diagnosing and staging syphilis beyond the current test options. A recent evaluation of a novel RPOCT IgA antibody test demonstrated the potential for a POCT for diagnosis of active syphilis but requires further evaluation (104).

## CONCLUSIONS

In specific settings, such as rural and remote areas, and among populations facing barriers to healthcare access, RPOCTs can be a crucial component of a strategy to combat the syphilis epidemic in HIC. Successful implementation requires strategic planning, community involvement, and ongoing monitoring to optimize program effectiveness. Given the current limitations of existing syphilis RPOCT, further research is needed on newer technologies to optimize the ability to accurately diagnose new and re-infections of syphilis.
